# A matter of scale: Identifying the best spatial and temporal scale of environmental variables to model the distribution of a small cetacean

**DOI:** 10.1002/ece3.70102

**Published:** 2024-08-06

**Authors:** Tiffany Goh, Mark Jessopp, Emer Rogan, Enrico Pirotta

**Affiliations:** ^1^ School of Biological, Earth and Environmental Sciences University College Cork, Enterprise Centre, Distillery Fields Cork Ireland; ^2^ MaREI Centre, Beaufort Building, Environmental Research Institute University College Cork Cork Ireland; ^3^ Centre for Research into Ecological and Environmental Modelling University of St Andrews St Andrews Fife United Kingdom

**Keywords:** environmental covariates, harbour porpoise, northeast Atlantic, species distribution models

## Abstract

The importance of scale when investigating ecological patterns and processes is recognised across many species. In marine ecosystems, the processes that drive species distribution have a hierarchical structure over multiple nested spatial and temporal scales. Hence, multi‐scale approaches should be considered when developing accurate distribution models to identify key habitats, particularly for populations of conservation concern. Here, we propose a modelling procedure to identify the best spatial and temporal scale for each modelled and remotely sensed oceanographic variable to model harbour porpoise (*Phocoena phocoena*) distribution within the Irish Exclusive Economic Zone. Harbour porpoise sightings were recorded during dedicated line‐transect aerial surveys conducted in the summers of 2016, 2021 and 2022. Binary generalised additive models were used to assess the relationships between porpoise presence and oceanographic variables at different spatial (5–40 km) and temporal (daily, monthly and across survey period) scales. Selected variables included sea surface temperature, thermal fronts, chlorophyll‐a, sea surface height, mixed layer depth and salinity. A total of 30,514 km was covered on‐effort with 216 harbour porpoise sightings recorded. Overall, the best spatial scale corresponded to the coarsest resolution considered in this study (40 km), while porpoise presence showed stronger association with oceanographic variables summarised at a longer temporal scale. Habitat models including covariates at coarse spatial and temporal scales may better reflect the processes driving availability and abundance of resources at these large scales. These findings support the hypothesis that a multi‐scale approach should be applied when investigating species distribution. Identifying suitable spatial and temporal scale would improve the functional interpretation of the underlying relationships, particularly when studying how a small marine predator interacts with its environment and responds to climate and ecosystem changes.

## Graphical abstract

In this study, we found that a multi‐scale approach should be applied when investigating appropriate scales when modelling species distribution. Identifying suitable spatial and temporal scale would improve the functional interpretation of the underlying relationships, particularly when studying how a small marine predator interacts with its environment and responds to climate and ecosystem changes.

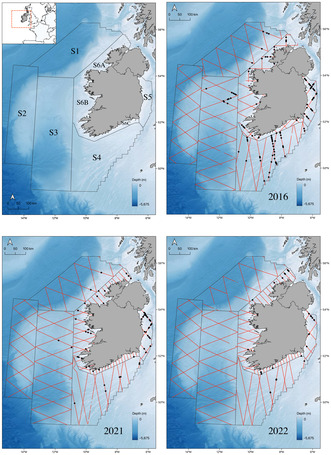

## 1 INTRODUCTION

Ecologists have long used species distribution models (SDMs) to assess the relationships between species occurrence and characteristics of their habitat through space and time (see review Guisan & Thuiller, 2005). Habitat modelling studies are critical to understand species' ecology and evolution, and to inform management and conservation efforts, particularly for identifying important areas for protected species (Embling et al., 2010; Liu et al., 2017; Penjor et al., 2021). The distribution of a species can be modelled as a function of a set of environmental variables, often derived from remotely sensed/modelled data or in situ observations (Becker et al., 2010; Graf et al., 2005; Putra & Mustika, 2020; Stuber & Fontaine, 2019). However, there is an ongoing debate on how to select the most appropriate spatial and/or temporal scales (also referred to as ‘resolution’) for the environmental variables included in a SDM (Guisan et al., 2007; Guisan & Thuiller, 2005; Levin, 1992; Mannocci et al., 2017; Moudrý et al., 2023; Scales et al., 2017; Wiens, 1989). The concept of species distribution being driven by environmental variables at various spatial and temporal scales was highlighted in Levin (1992). It has been suggested that there is no single resolution at which species–habitat associations should be studied, as species may respond to different ecological processes at a range of scales (Wiens, 1989), particularly when they are wide‐ranging (Scales et al., 2017). For example, predators are expected to range widely and, therefore, select habitat at broader scales than those of their prey (Hostetler & Holling, 2000). As a result, predator distribution follows the hierarchical patch structure of their prey, thus responding to complex heterogeneity at several scales (Miller et al., 2015; Redfern et al., 2006). In the current era of climate uncertainty and accelerating biodiversity loss, it is imperative to assess the importance of these scales and to monitor how their importance may change with rapid ecosystem alteration occurring globally (Malhi et al., 2020). For instance, climate change influences local conditions (e.g. causing rising temperatures) but may also impact the broader community, with resulting consequences for a given species at various spatial and temporal scales (Harley et al., 2006). Considering environmental associations at different scales in SDMs is pivotal to understanding what processes drive a species' distribution and to assess potential habitat availability and loss (Guisan & Thuiller, 2005; Martínez et al., 2012).

SDMs are frequently developed using predictor variables at a single scale (McGarigal et al., 2016). Generally this corresponds to the finest spatial resolution available, which is assumed to allow for more accurate representation of how species interact with localised environmental conditions (Liu et al., 2017; Riekkola et al., 2019; Soberón, 2007; Stephenson et al., 2020). However, using fine‐scale predictors may also be misleading if animals were only travelling through an area. The selection of appropriate spatio‐temporal scales in SDMs has been studied across many terrestrial and marine species (Becker et al., 2010; Gottschalk et al., 2011; Graf et al., 2005; Levin, 1992; Mateo‐Sánchez et al., 2016; Redfern et al., 2006; Stuber & Fontaine, 2019; Wiens, 1989). For example, Gottschalk et al. (2011) examined the effects of different spatial scales of a land‐use map (from 1 to 1000 m) on predicting bird distribution and found that the occurrence of different bird species was better predicted using models with different spatial resolutions. A study by Graf et al. (2005) on a forest grouse species (*Tetrao urogallus*) compared single spatial scale habitat models with a multi‐scale model (1 to 1100 ha) and found that the latter performed better. Likewise, brown bears (*Ursus arctos*) in the Cantabrian Range in Spain responded to environmental factors at different spatial scales (0.25–64 km) across seasons and time periods, suggesting the influence of processes underlying spatial and temporal variations in habitat use (Mateo‐Sánchez et al., 2016).

In the marine realm, one study determined that the encounter rate of nine cetacean species was best predicted using satellite‐derived sea surface temperature at the coarsest available spatial resolution (~33 km), while for one species it was better captured using an intermediate resolution of ~17 km (Becker et al., 2010). However, these authors also found that models built using finer temporal resolution resulted in better predictive ability (Becker et al., 2010). Similarly, a study by Fernandez et al. (2018) demonstrated that ecological niche models of ten cetacean species using 8‐day means of environmental variables outperformed models with monthly resolutions. Additionally, multi‐scale analyses on the habitat preference of Azorean blue whales (*Balaenoptera musculus*) revealed that coarse spatial resolution models (50 km) were better fitting compared to 1 km spatial resolution models (González García et al., 2018). These authors also found that a 1 km spatial resolution model with weekly composites of satellite‐derived oceanographic variables performed better than the fine spatial resolution daily model, due to frequent cloud coverage resulting in many missing values in fine resolution data (González García et al., 2018). When using oceanographic variables in marine SDM studies, the selection of an appropriate spatial and temporal scale for the environmental variables is complicated by the mismatch between the spatial and temporal distribution of primary production and the distribution of secondary and tertiary production (Jaquet & Whitehead, 1996; Vinogradov, 1981). For example, peaks in primary productivity may be spatially and temporally separated from peaks in secondary production due to lags caused by advection processes associated with currents (Dragon et al., 2010; Guinet et al., 2001). The inclusion of relevant scales that correspond to known features of a species' habitat is, therefore, paramount in the development and interpretation of SDMs, but this may be difficult to determine a priori. The selection of appropriate scales should be informed by the purpose of the study, be it to describe patterns (exploratory), test hypotheses (inferential) or forecast future distribution (predictive) (Tredennick et al., 2021). Considering the ecological question examined is also important (Dungan et al., 2002; Mannocci et al., 2017).

Mannocci et al. (2017) discussed three different ecological scales relevant for modelling species distribution of highly mobile marine animals: fine‐scale, mesoscale and macroscale. Fine‐scale studies describe relationships between species and ephemeral oceanographic processes over areas less than 10 km, which act as proxies of prey aggregation into patches that are targeted by highly mobile marine animals (Benoit‐Bird & Au, 2003; Fauchald et al., 2000; Torres et al., 2008). Mesoscale analyses highlight associations of species with processes such as eddies and fronts, which can extend over tens to hundreds of kilometres and persist for hours to months (Becker et al., 2010; Cox et al., 2018). These associations can reveal the use of large areas in the open ocean by migratory animals to source for biologically rich foraging opportunities (Becker et al., 2010). Finally, macroscale studies relate species occurrence with oceanographic processes over thousands of kilometres and spanning many years. These analyses often investigate the geographic ranges of species and associate them with processes that have taken place over evolutionary timescales (Torres et al., 2013).

Harbour porpoises (*Phocoena phocoena*) are among the most abundant cetacean species in the European Atlantic and are generally distributed in coastal and shelf waters (Hammond et al., 2002, 2013). This small cetacean species has high energetic requirements (Wisniewska et al., 2016) and a limited capacity to store energy in blubber (Koopman et al., 2002). Therefore, harbour porpoise movement patterns are likely to be strongly associated with the distribution of their prey. As a top predator, harbour porpoises act as an important indicator species of multitrophic ecosystem change as they integrate ecological signals over various spatial and temporal scales (Gilles et al., 2016; Hammond et al., 2013; Kiszka et al., 2015). They are a species exposed to major anthropogenic threats such as incidental capture in fishing gear (Bjørge et al., 2013), pollution (Murphy et al., 2010) and underwater noise from pile‐driving during offshore developments (Pirotta, Brookes, et al., 2014). Consequently, harbour porpoises are listed in the EU Habitats Directive Annex II and IV (EU‐COM, 1992) and on the OSPAR list of threatened and declining species and habitats of the Northeast Atlantic (OSPAR, 2008). Between 1994 and 2005, an observed southern re‐distribution of harbour porpoises was reported in the northeast Atlantic (Hammond et al., 2013). The abundance of harbour porpoises in the North Sea has been relatively stable since 2005 but the number of sightings in the English Channel is progressively increasing (Gilles et al., 2023). However, preliminary analyses suggest that the abundance of porpoises in the Celtic and Irish Seas may be in decline since 2009 (NAMMCO & IMR, 2019). In this study, we aimed to develop a modelling procedure to identify the best spatial and temporal scale (focusing on fine‐ and mesoscale) of six modelled and remotely sensed oceanographic variables. We hypothesise that harbour porpoises respond to oceanographic conditions over multiple spatial and temporal resolutions. If there is a scale effect, accounting for scale will enhance the development of predictive SDMs that can provide a more accurate representation of how a small marine predator interacts with its environment and potentially responds to climate and ecosystem changes.

## 2 MATERIALS AND METHODS

### 2.1 Survey design

**FIGURE 1:**
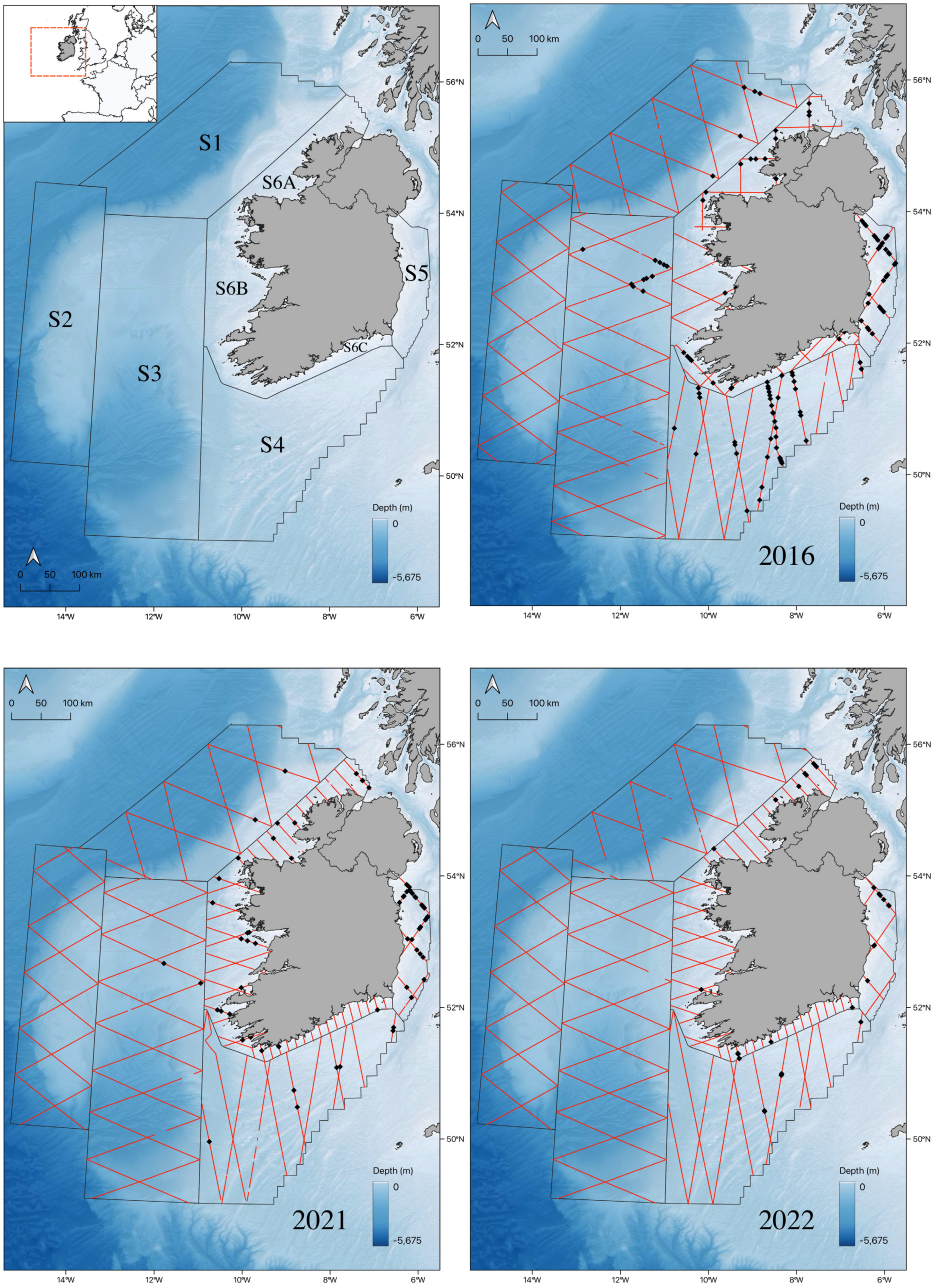
Harbour porpoise sightings (black dots) and effort (red lines) for 2016, 2021 and 2022. Top left map shows Strata 1‐6C of the ObSERVE aerial surveys. Bathymetry data were from the General Bathymetric Chart of the Oceans (GEBCO, https://www.bodc.ac.uk/data/published_data_library/catalogue/10.5285/c6612cbe‐50b3‐0cff‐e053‐6c86abc09f8f/).

Harbour porpoise sightings were recorded during the ObSERVE aerial survey programme conducted within the Irish EEZ (Exclusive Economic Zone) between late May and Sept 2016 (Rogan et al., 2018), 2021 and 2022. The study area was divided into eight strata: stratum 1 to stratum 5 covered offshore areas and the Irish Sea, and stratum 6A, 6B and 6C comprised inshore waters along the north, west and south coasts of Ireland, respectively (Figure 1). Within each stratum, two equally spaced zig‐zag survey tracks were surveyed, which were designed to ensure equal coverage probability, with the exception of stratum 6A and 6C where parallel transects were used in 2021 and 2022.

### 2.2 Aerial survey methods

Survey tracks were flown at a target altitude of 183 m and a ground speed of 167 km h^−1^ (90 knots), in Beaufort sea state 3 or less and with good visibility of 1 km or more.

In 2016, surveys were conducted using a Britten‐Norman 2 Islander, while in 2021 and 2022 surveys used a Partenavia P68. Both aircraft were fixed high‐wing, twin‐engine aircraft equipped with bubble windows to allow observations directly underneath the plane. In addition to the pilot, the survey team comprised at least three trained observers, two of whom undertook observations from both sides of the aircraft while a navigator entered real‐time environmental and sightings data into a laptop computer. Observers rotated through these positions after each flight to avoid potential biases in data collection and to reduce navigator and observer fatigue.

A data logging software linked to GPS recorded positional information every 2 s during the surveys in 2016. An upgraded version of the software ‘SAMMOA’ (SAMMOA, 2022) was utilised during the 2021/2022 surveys and logged the aircraft's position every second. The following environmental conditions were recorded at the beginning of each transect and whenever conditions changed while on survey: Beaufort sea state, turbidity (determined visually; 0 – clear water to 3 – very turbid), rain (yes or no), glint and glare severity (0 – no glare to 3 – severe), cloud coverage and an overall subjective assessment of sighting conditions (good, moderate or poor, in relation to the probability of seeing harbour porpoises).

Surveys followed standard line‐transect methodology for aerial surveys (Buckland et al., 2001). Observers recorded sightings in a search area extending out to 500 m from the survey line on each side of the aircraft. For each harbour porpoise sighting, the group size and declination angle (measured with an inclinometer) to the group when they came abeam were recorded. Using the altitude of the aircraft, perpendicular distances were then calculated from the angles (Buckland et al., 2001).

### 2.3 Detection function modelling

Multiple covariate distance sampling (MCDS) models were used to model the detection probability of harbour porpoises in each year (Marques & Buckland, 2004). The MCDS method is an extension of conventional distance sampling (Buckland et al., 2001), where the detection function is modelled as a function of both perpendicular distance and one or more covariates. Hazard rate and half‐normal models were first fitted to the data and the model with lowest Akaike Information Criterion (AIC) was retained before testing the influence of covariates (Buckland et al., 2001). Goodness‐of‐fit testing, quantile‐quantile plots and Cramer‐von Mises test results were also assessed for each model. Subsequently, a single covariate was added to the detection function, including sea state, glare, turbidity, cloud coverage, group size and observer. If the inclusion of a covariate resulted in reduction of AIC greater than two units, that covariate was retained in the detection function and the effective strip width (ESW) was estimated accordingly. Harbour porpoise sightings at extreme distances within the 500 m survey line were not truncated and multiple covariates were not concurrently included in the detection function model due to the low number of observations (2016: 117, 2021: 67 and 2022: 32). MCDS analyses were conducted in R (version 4.2.2, RStudio 2022.12.0 + 353, R Core Team, 2022) using the ‘distance’ package (version 1.0.7, Miller et al., 2019).

### 2.4 Data processing for habitat modelling

Harbour porpoise sightings and survey effort data were associated to the centroids of 4 km × 4 km grid cells, similar to the finest resolution of the environmental variables used in this study (Figures S6–S11) and to minimise cells with zero survey effort (Figures S12–S14). Sightings were converted to binary presence or absence data within each cell for each survey period. Survey effort was calculated using the ESW derived from the detection function. During periods where monitoring was carried out only on one side of the plane (due to differences in environmental conditions on the two sides of the aircraft), a separate ESW was calculated to account for the halved effort. QGIS 3.22.10 was used for data processing and the resulting datasets were then merged across years and imported into R for all subsequent analysis.

### 2.5 Environmental variables

Modelled and remotely sensed oceanographic data covering the study area were acquired from the E.U. Copernicus Marine Service Information website (Level 4 products–https://data.marine.copernicus.eu/). We obtained daily values of sea surface temperature (SST), chlorophyll‐a concentration (CHLA), salinity (SAL), mixed layer depth (MLD) and sea surface height (SSH), providing proxies of oceanographic processes potentially associated with prey aggregation and abundance. In addition, the occurrence of thermal fronts (Fronts) was calculated as the slope of SST (SST gradient computed according to Horn, 1981), with high slope values suggesting the potential occurrence of a front. To determine which spatio‐temporal resolution of these environmental variables better predicted harbour porpoise distribution, three spatial (5, 20 and 40 km) and three temporal (daily, monthly and average across survey period) resolutions were considered (Table 1).

**TABLE 1:** Spatial and temporal scales of environmental variables used in harbour porpoise habitat modelling.

Environmental variables	Year	Original resolution	Spatial resolution (km)	Temporal resolution	Data source	Source information
*Physiographic*	–	30 arc‐second	–	–	–	MARSPEC (http://marspec.org/)
Depth (m)						
*Oceanographic*						Copernicus Marine Service (https://data.marine.copernicus.eu/)
Sea surface temperature (°C)	2016	0.05° × 0.05° (~5 km)	5, 20, 40	Daily, monthly, survey period	Satellite observations	European North West Shelf/Iberia Biscay Irish Seas—High Resolution L4 Sea Surface Temperature Reprocessed
	2021	0.02° × 0.02° (~2 km)	5, 20, 40	Daily, monthly, survey period	Satellite observations	European North West Shelf/Iberia Biscay Irish Seas—High Resolution ODYSSEA L4 Sea Surface Temperature Analysis
	2022	0.02° × 0.02° (~2 km)	5, 20, 40	Daily, monthly, survey period	Satellite observations	European North West Shelf/Iberia Biscay Irish Seas—High Resolution ODYSSEA L4 Sea Surface Temperature Analysis
Thermal fronts (°C) Derived from slope of SST	2016	0.05° × 0.05° (~5 km)	5, 20, 40	Daily, monthly, survey period	Satellite observations	European North West Shelf/Iberia Biscay Irish Seas—High Resolution L4 Sea Surface Temperature Reprocessed
	2021	0.02° × 0.02° (~2 km)	5, 20, 40	Daily, monthly, survey period	Satellite observations	European North West Shelf/Iberia Biscay Irish Seas—High Resolution ODYSSEA L4 Sea Surface Temperature Analysis
	2022	0.02° × 0.02° (~2 km)	5, 20, 40	Daily, monthly, survey period	Satellite observations	European North West Shelf/Iberia Biscay Irish Seas—High Resolution ODYSSEA L4 Sea Surface Temperature Analysis
Chlorophyll‐a concentration (mg m^−3^)	2016	0.111° × 0.067° (~7 km)	5, 20, 40	Daily, monthly, survey period	Numerical models	Atlantic‐ European North West Shelf‐ Ocean Biogeochemistry Reanalysis
	2021	0.111° × 0.067° (~7 km)	5, 20, 40	Daily, monthly, survey period	Numerical models	Atlantic‐ European North West Shelf‐ Ocean Biogeochemistry Reanalysis
	2022	0.111° × 0.067° (~7 km)	5, 20, 40	Daily, monthly, survey period	Numerical models	Atlantic—European North West Shelf—Ocean Biogeochemistry Analysis and Forecast
Sea surface height (m)	2016	0.111° × 0.067° (~7 km)	5, 20, 40	Daily, monthly, survey period	Numerical models	Atlantic‐ European North West Shelf‐ Ocean Physics Reanalysis
	2021	0.111° × 0.067° (~7 km)	5, 20, 40	Daily, monthly, survey period	Numerical models	Atlantic‐ European North West Shelf‐ Ocean Physics Reanalysis
	2022	0.111° × 0.067° (~7 km)	5, 20, 40	Daily, monthly, survey period	Numerical models	Atlantic—European North West Shelf—Ocean Physics Analysis and Forecast
Mixed layer depth (m)	2016	0.111° × 0.067° (~7 km)	5, 20, 40	Daily, monthly, survey period	Numerical models	Atlantic‐ European North West Shelf‐ Ocean Physics Reanalysis
	2021	0.111° × 0.067° (~7 km)	5, 20, 40	Daily, monthly, survey period	Numerical models	Atlantic‐ European North West Shelf‐ Ocean Physics Reanalysis
	2022	0.111° × 0.067° (~7 km)	5, 20, 40	Daily, monthly, survey period	Numerical models	Atlantic—European North West Shelf—Ocean Physics Analysis and Forecast
Salinity (PSU)	2016	0.111° × 0.067° (~7 km)	5, 20, 40	Daily, monthly, survey period	Numerical models	Atlantic‐ European North West Shelf—Ocean Physics Reanalysis
	2021	0.111° × 0.067° (~7 km)	5, 20, 40	Daily, monthly, survey period	Numerical models	Atlantic‐ European North West Shelf—Ocean Physics Reanalysis
	2022	0.111° × 0.067° (~7 km)	5, 20, 40	Daily, monthly, survey period	Numerical models	Atlantic—European North West Shelf—Ocean Physics Analysis and Forecast

Values for all environmental variables were associated with harbour porpoise presence/absence data using the ‘raster’ package (Hijmans & van Etten, 2012). To generate coarser spatial resolutions (20 and 40 km), spatial aggregation was applied (Hijmans & van Etten, 2012). The ‘aggregate’ function in the ‘raster’ package for R can be used to reduce the resolution of a raster by averaging the values of multiple contiguous cells. Environmental variables with an original resolution of 5 km consist of rasters of 5 × 5 km cells. To aggregate these rasters to a 20‐ or a 40‐km spatial resolution, the aggregating factors were set to 4 and 8, respectively. The aggregating factors for environmental variables with an original raster resolution of 7 km were set to 3 and 6, resulting in spatial resolutions of approximately 20 and 40 km, respectively. Monthly and average values for each survey period were calculated as the mean of daily values for each month, and the mean of daily values within a survey period, respectively. Further details of the spatial and temporal scales of the modelled and remotely sensed data are reported in Table 1. In addition to oceanographic variables, we also included depth, obtained from the MARSPEC dataset within the ‘sdmpredictors’ package version 0.2.14, at a 30 arc‐second resolution (Sbrocco & Barber, 2013).

Separate datasets were compiled for each oceanographic covariate (i.e. excluding depth). Before fitting the habitat models, data points with missing covariate values were excluded and chlorophyll‐a and mixed layer depth datasets were log‐transformed to reduce the variability of the data (Figures S1 and S2).

### 2.6 Habitat modelling

#### 2.6.1 Model development

We used Generalised Additive Modelling (GAM) with thin plate regression splines to assess the relationships between harbour porpoise presence and the environmental variables at different spatio‐temporal scales. This data‐driven approach has been extensively used in the study of cetacean distribution as it can accommodate non‐parametric, smooth relationships with predictor variables (Hastie & Tibshirani, 1990). Binary GAMs with a logit link function were fitted in R using the ‘mgcv’ package version 1.8‐41 (Wood, 2017). Although the estimated splines allow for higher predictive power than linear models, they incur the risk of overfitting the data, resulting in complex non‐linear relationships that may not be ecologically meaningful (Austin, 2007). Therefore, we imposed a greater penalty on ‘wiggliness’, using a gamma value of 1.4 over the default of 1 (Wood, 2017).

First, we developed a base model including year of survey, effort and depth. Subsequently, we tested the inclusion of each oceanographic variable at each combination of spatial and temporal resolution (e.g. sst5d: SST at 5 km spatial and daily resolution; sst20m: SST at 20 km spatial and monthly resolution; sst40p: SST at 40 km spatial resolution and summarised over the survey period). The general structure of the model was:

$$\log\left(\frac{Ρ\left(Y=1\right)}{1-Ρ\left(Y=1\right)}\right)=\beta_{0}+\beta_{1}X_{1}+f_{2}\left(X_{2}\right)+f_{3}\left(X_{3}\right)+f_{4}\left(X_{4}\right),$$

where $\log\left(\frac{Ρ\left(Y=1\right)}{1-Ρ\left(Y=1\right)}\right)$ is the logit function that links the probability of sighting a porpoise in a cell, $Ρ\left(Y=1\right)$, to the linear predictor, $\beta_{0}$ is the intercept, $\beta_{1}X_{1}$ is a parametric term for the effect of year, and $f_{2}\left(X_{2}\right),f_{3}\left(X_{3}\right),f_{4}\left(X_{4}\right)$ are the smooth functions of effort, depth and one of the oceanographic variables at a given spatio‐temporal resolution. All explanatory variables excluding year were standardised prior to fitting the models.

#### 2.6.2 Model selection and validation

Model selection was carried out using splines with shrinkage, which penalises the null space and can thus shrink irrelevant terms to zero (Marra & Wood, 2011). The final model for each oceanographic variable was the one with the lowest AIC, but models within 2 AIC units of the best model were also considered and discussed. Multicollinearity and concurvity between the variables in the base and final models were checked to identify potential issues with the interpretation of the shape of the estimated splines. The variance inflation factor (VIF) was calculated using the ‘car’ package (Fox & Weisberg, 2019) and concurvity estimates were derived using the ‘mgcv’ package (Wood, 2017). If a variable had VIF ≥2 or exceeded a concurvity value of 0.8, an issue with the base model would be flagged.

Using the ‘pROC’ package (Robin et al., 2011), the predictive power of each model was examined using the Area Under the Curve (AUC of the Receiving Operating Characteristic (ROC) curve), where a value close to 1 indicated high discriminatory power (Beck & Shultz, 1986). Additionally, an optimal classification threshold was selected from the ROC curve, using cross‐validation to maximise the true presence and absence rates (Fielding & Bell, 1997). This optimal threshold was used to build a confusion matrix to summarise the goodness‐of‐fit of the model (Fielding & Bell, 1997). The confusion matrices compared binary predictions to the observed values for each oceanographic dataset and were constructed using the ‘caret’ package (Kuhn, 2008). From the confusion matrix, we derived the overall *accuracy* (proportion of correctly classified presences and absences), *sensitivity* (proportion of true presences) and *specificity* (proportion of true negatives).

## 3 RESULTS

### 3.1 Survey effort

A total of 30,514 km was covered within the Irish EEZ during aerial surveys conducted over three summers, with comparable effort in each of the sampling years (2016: 10,010 km, 2021: 10,200 km, 2022: 10,304 km). In 2016, there were 117 sightings of harbour porpoises, comprising 161 animals and the majority of these sightings were recorded in Beaufort sea state 1. A decrease in harbour porpoise observations was noted in 2021 and 2022, with 67 sightings of 86 individuals and 32 sightings of 41 animals, respectively. Most of the 2021 sightings were recorded in Beaufort sea state 2, while in 2022 they were predominately recorded in Beaufort sea state 1. Out of the 216 porpoise sightings, 133 observations were of single individuals. There was also limited variability in group size (mean ± SD: 1.5 ± 0.25). Moreover, out of the 10,422 grid cells that were surveyed in this study, porpoises were detected only in 192 cells. Given these features, we, therefore, decided to use a simpler binary analysis, rather than modelling our data as counts. Overall, harbour porpoises were concentrated on the continental shelf within the strata in the Irish Sea and along the south coast of Ireland (Figure 1, Strata 4–6).

### 3.2 Detection function modelling

The hazard rate was the best‐fitting detection function model for 2016 and 2021, whereas in 2022 a half‐normal function was preferred (Figures S3–S5). There was no support for the inclusion of environmental covariates recorded on survey in the detection functions; therefore, a standard ESW of 168, 172 and 222 m was used for 2016, 2021 and 2022, respectively. The Cramer‐von Mises goodness‐of‐fit test probabilities ranged from 0.45 to 0.90, indicating a good fit of the models to the data.

### 3.3 Habitat modelling

#### 3.3.1 Model selection

**FIGURE 2:**
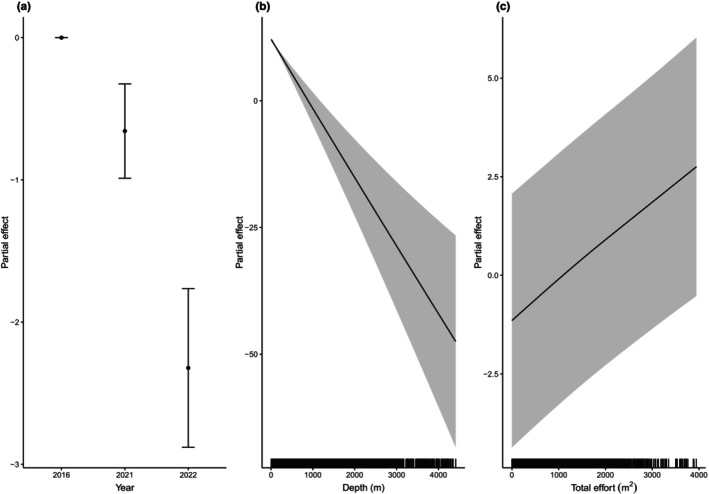
Partial effects of (a) year, (b) depth and (c) effort in the base model on the probability of harbour porpoise presence in a cell. A rug plot in the plots for the effects of depth and effort shows the range of values for each covariate, and the shaded areas denote the 95% confidence intervals. All partial effects plots are untransformed and unscaled.

The best base model included year, depth and effort. The estimated splines did not shrink to 0 and all variables had a significant effect on the probability of sighting a harbour porpoise (*p* < .05, Figure 2). The probability of sighting a porpoise was highest in waters <200 m depth and with increasing effort (Figure 2b,c). The model also captured the decrease in harbour porpoise presence during the 3 years of survey (Figure 2a). The area under the ROC curve was 0.89, indicating high predictive power. Multicollinearity and concurvity scores did not highlight any issue.

**FIGURE 3:**
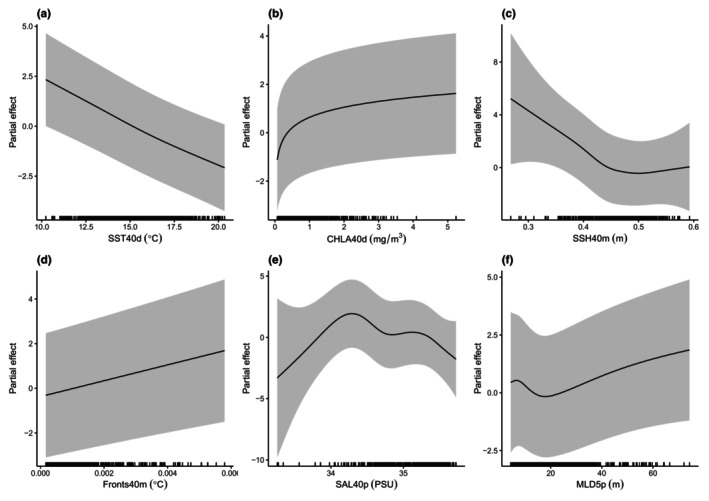
Partial effects of the oceanographic covariates used to model the probability of harbour porpoise presence in a cell. We plot the estimated spline relationships between harbour porpoise presence and the selected spatio‐temporal resolution for (a) sea surface temperature (SST), (b) chlorophyll‐a (CHLA), (c) sea surface height (SSH), (d) thermal fronts (Fronts), (e) salinity (SAL) and (f) mixed layer depth (MLD). Oceanographic variables were selected at a spatial resolution of 5 or 40 km, and at a *d* = daily, *m* = monthly or *p* = survey period temporal resolution. Rug plots show the range of values for each covariate, and the shaded areas denote the 95% confidence intervals. All partial effects plots are untransformed and unscaled.

The spatio‐temporal resolutions of each oceanographic variable that best predicted harbour porpoise presence is detailed in Table 2 and Table S1. Overall, the coarsest spatial resolution was preferred across variables, with the exception of MLD (Figure 3). The preferred temporal resolution was variable, with models performing best when SST and CHLA were included at a daily resolution, SSH and Fronts at a monthly resolution, and SAL and MLD at the temporal resolution of the survey period (Figure 3). Multiple resolutions for CHLA, MLD and Fronts resulted in comparable models (i.e. within two units of AIC of each other). For CHLA, two models were selected, with a 40 km spatial resolution and either a daily (AIC = 1437.6) or a monthly temporal resolution (AIC = 1439.3). For MLD, comparable models were obtained using (i) 5 km spatial resolution and a temporal resolution of the survey period (AIC = 1453.2), (ii) 5 km spatial resolution and a daily temporal resolution (AIC = 1454.5) and (iii) 20 km spatial resolution and a daily temporal resolution (AIC = 1455.8). The top three models for Fronts all included a monthly temporal resolution, but either a 40 km (AIC = 1356.6), a 20 km (AIC = 1356.7) or a 5 km spatial resolution (AIC = 1357.9). The deviance explained by the final models were higher compared to base models for each oceanographic variable and ranged from 24.2% to 26.5%, with AUC scores of 0.9 across variables (Table 2). When comparing model performance of the best spatio‐temporal scale for each covariate to the worst fitting scale, the deviance explained for all variables decreased by up to 2.6% (Table S2). Furthermore, there was no significant effect of MLD and SSH on harbour porpoise presence in a cell when the worst fitting spatio‐temporal scale was used (*p* > .05, Table S2).

**TABLE 2:** Comparison between final and base models for each oceanographic variable and associated goodness‐of‐fit measures.

	VIF	Concurvity	*p*‐value of smooth terms	Deviance explained (%)	Area under ROC curve	Confusion matrix statistics		
						Accuracy (%)	Sensitivity (%)	Specificity (%)
SST40d	1.0	0.66	<.0001	25.8	0.9	76	75	93
Depth	1.0	0.20	<.0001					
Effort	1.0	0.33	<.0001					
Base model								
Depth	1.0	0.01	<.0001	22.6	0.89	75	75	91
Effort	1.0	0.33	<.0001					
CHLA40d	1.0	0.52	<.0001	24.7	0.9	77	77	90
Depth	1.0	0.13	<.0001					
Effort	1.0	0.33	<.0001					
Base model								
Depth	1.0	0.01	<.0001	23.1	0.89	76	76	90
Effort	1.0	0.33	<.0001					
SSH40m	1.1	0.39	<.0001	25.4	0.9	77	77	92
Depth	1.1	0.24	<.0001					
Effort	1.0	0.33	<.0001					
Base model								
Depth	1.0	0.01	<.0001	23.1	0.89	76	76	90
Effort	1.0	0.33	<.0001					
Fronts40m	1.1	0.68	<.0001	25.1	0.9	78	77	92
Depth	1.1	0.37	<.0001					
Effort	1.0	0.34	<.0001					
Base model								
Depth	1.0	0.02	<.0001	24.2	0.9	73	73	95
Effort	1.0	0.34	<.0001					
SAL40p	1.2	0.68	<.0001	26.5	0.9	78	78	90
Depth	1.2	0.67	<.0001					
Effort	1.0	0.33	<.0001					
Base model								
Depth	1.0	0.01	<.0001	23.1	0.89	76	76	90
Effort	1.0	0.33	<.0001					
MLD5p	1.2	0.54	<.0001	24.2	0.9	75	74	92
Depth	1.2	0.48	<.0001					
Effort	1.0	0.33	<.0001					
Base model								
Depth	1.0	0.01	<.0001	23.1	0.89	76	76	90
Effort	1.0	0.33	<.0001					

*Note*: Final models include the spatio‐temporal resolution that best predicted the probability of harbour porpoise presence.

Abbreviations: ROC, receiving operating characteristic; VIF, variance inflation factor; 40d, 40 km spatial and daily temporal resolution; 40m, 40 km spatial and monthly temporal resolution; 40p, 40 km spatial and survey period temporal resolution; 5p, 5 km spatial and survey period temporal resolution.

All oceanographic covariates in the final models had a significant effect on the probability of harbour porpoise presence (*p* < .05). The probability of harbour porpoise presence was highest at 10°C, but declined in warmer waters (>15°C) and with increasing SSH (Figure 3a,c). For increasing CHLA, Fronts and MLD, the probability of harbour porpoise presence also increased (Figure 3b,d,f). Finally, the probability of harbour porpoise presence was highest when salinity ranged between 34 and 34.5 PSU (Figure 3e).

## 4 DISCUSSION

This study proposes a modelling procedure to identify the best spatial and temporal scale of six commonly used modelled and remotely sensed oceanographic variables to model harbour porpoise distribution within the northeast Atlantic. Models were built using systematic data collected over 3 years through aerial surveys conducted in summer months. Overall, the best spatial scale corresponded to the coarsest resolution considered in this study, while the best temporal resolution was more variable. However, harbour porpoise presence showed the strongest association with environmental predictors of a coarser temporal scale. These results support the hypothesis that harbour porpoises respond to oceanographic conditions over multiple spatial and temporal scales, and highlight the importance of scale when modelling species distribution using a combination of modelled and remotely sensed oceanographic variables.

### 4.1 Spatial and temporal scales

SDMs environmental predictors were selected at the coarsest spatial resolution (40 km), matching the large scale of the survey data collected. Sightings data and oceanographic observations collected over large geographical areas tend to be better modelled using variables at coarser rather than fine scales (Mannocci et al., 2017), as coarse resolutions may better reflect the processes driving the abundance of prey available to upper trophic level predators distributed over a wider space. For example, in an 818,000 km^2^ study area off California, Becker et al. (2010) reported that the best predictive ability from cetacean habitat models was obtained using the coarsest SST spatial resolution (33.3 km). Conversely, fine‐scale distribution of bottlenose dolphin (*Tursiops truncatus*) prey patches in Florida bay were best elucidated using environmental predictors at a 50 m spatial resolution, due to the extreme heterogeneity of this habitat (Torres et al., 2008). Aerial surveys of cetaceans provide a snapshot in time of the dynamic distribution of highly mobile species. In addition, cetaceans spend most of their time underwater, leading to infrequent and dispersed of observations. The coarse nature of the data collection, combined with the small number of harbour porpoise sightings in our surveys, makes it challenging to identify the processes that drive the species' fine‐scale habitat use and may have contributed to the selection of the coarser spatial scales in our models. In general, these data limitations likely apply across many species of marine megafauna, suggesting that coarse‐scale spatial covariates may be more appropriate to represent their underlying spatial use pattern.

The dynamic nature of marine ecosystems means that environmental resources are often heterogeneously distributed in time, as well as in space. Although the influence of spatial scale on the outcomes of SDMs have long been recognised among ecological modellers, few studies have explicitly explored the influence of temporal resolution (Becker et al., 2010; Mannocci et al., 2014; Pirotta, Thompson, et al., 2014; Scales et al., 2017). Significant periodicities of physical processes can be observed from daily to annual and even multi‐annual scales (Mannocci et al., 2017). Here, we found that harbour porpoise presence was most associated with longer time scale oceanographic predictors (monthly and average across survey period), perhaps in association with persistent oceanographic features (Mannocci et al., 2014; Palacios et al., 2006; Scales et al., 2014). Seasonal mean maps of fronts and CHLA are used to represent physical and biological environments and can be used to identify persistent features where marine predators congregate (Palacios et al., 2006). A study on northern gannets (*Morus bassanus*) found that the species was more likely to respond to seasonally persistent frontal zones compared to weekly front composites, suggesting a preference for more predictable profitable foraging grounds (Scales et al., 2014). Similarly, Mannocci et al. (2014) also demonstrated that cetaceans and seabirds in the Southwest Indian Ocean responded to persistent oceanographic features inferred from seasonal (average across survey period) and climatological (long‐term) temporal resolution compared to a weekly resolution.

One benefit of incorporating longer time scale modelled or remotely sensed composites such as seasonal and climatological data fields is that the data are less affected by cloud coverage, especially at higher latitudes. Indeed, environmental data of coarse spatial and temporal resolution have been useful in building habitat models and mapping distributions of wide‐ranging marine predators (Kaschner et al., 2006; Mannocci et al., 2014). From a functional perspective, physical processes such as the transport of planktonic organisms may result in temporal lags between the oceanographic variables that are included in SDMs and the availability or abundance of prey resources targeted by marine predators. Time lags have been documented between CHLA peaks and zooplankton blooms, and, in turn, between zooplankton blooms and aggregations of marine predators. For example, Littaye et al. (2004) compared three temporal scales for CHLA, related to primary production processes and found that fin whale (*Balaenoptera physalus*) summer distribution in the northwestern Mediterranean Sea was associated with spring primary production with a time lag of a few weeks. Furthermore, harbour porpoise densities in the German Bight were highest in areas of low nitrogen concentration, which could indicate the onset of secondary production succeeding a phytoplankton bloom (Gilles et al., 2011). In our study area, a seasonal geostrophic jet flows parallel to the coast in the summer as part of the Irish Coastal Current system, transporting plankton around the coast from the south of Ireland in a clockwise movement (Raine et al., 2010). Off the west coast of Ireland, the current system is driven by water flows from the Irish Coastal Current, the North Atlantic Current and the Irish Shelf Current (Holliday et al., 2020; Raine et al., 2010; Reid et al., 2003). Additionally, seasonal stratification occurs during summer months, posing a key barrier to the replenishment of nutrients from deep cold waters to surface waters (O'Boyle & Nolan, 2010). Here, habitat models selecting for a temporal resolution averaged over longer time scales were preferable as this integrates information on the time lags and may be more representative of the underlying processes occurring in the study area.

The combination of the dynamic nature of oceanic conditions, the scale at which animal observations are recorded and the resolution of environmental data influences model inference (Scales et al., 2017). In this study, the base model without oceanographic variables showed a good performance at capturing harbour porpoise distribution (Table 2). The inclusion of oceanographic variables led to a small improvement in the goodness‐of‐fit of the model, but importantly it provided additional insights into the potential processes that drive the species' distribution. Across all variables, we also found higher deviance explained for the best spatio‐temporal scale model compared to the worst fitting scale, and, in the case of SSH and MLD, relationships were not significant when the worst fitting spatio‐temporal scale was used (Table S2). Therefore, the identification of the best spatial and temporal scale for each environmental variable is critical for the correct functional interpretation of underlying relationships between harbour porpoise and its habitat. The selected spatio‐temporal scales may have been different in different contexts where other fine‐scale processes are associated with porpoise habitat use. For example, in the Bay of Fundy, fine‐scale tidally driven oceanographic features aggregate prey, increasing prey availability for harbour porpoises in the summer (Johnston et al., 2005).

### 4.2 Other modelling considerations

Both modelled and remotely sensed oceanographic data are readily accessible to ecologists studying the relationships between species and their environment. The modelled environmental data used in this study are subject to stringent validation (a Quality Information Document and a Synthesis Quality Overview are available from E.U. Copernicus Marine Service Information, at https://data.marine.copernicus.eu/). High‐quality modelled data can be useful to fill gaps in areas with limited data coverage, whether from satellite or in situ observations. However, model products introduce an additional source of uncertainty in the analysis, and efforts should continue to test the accuracy of oceanographic model predictions in different regions. For example, modelled chlorophyll data used in this study correctly captured broad scale patterns over the North‐West European Shelf waters inferred from satellite estimates, but underestimated chlorophyll concentration on the continental shelf when compared to in situ data, particularly along the English Channel and the North Sea (Gutknecht et al., 2019). This bias was associated with nutrients from river inputs causing a sharp increase in chlorophyll, which the model failed to predict due to lack of observational data to constrain the boundaries of the bloom (Gutknecht et al., 2019).

The observed differences in detection curves and effective strip width across the years could not be attributed to the any of the covariates we tested. However, a relatively small sample size can lead to higher variability in the detection function, making it difficult to accurately assess the importance of potential explanatory variables (Buckland et al., 2001). Moreover, population changes, such as fluctuations in the number of harbour porpoises or their distribution, could also have affected detection rates from year to year (Buckland et al., 2015). Finally, other environmental variables that were not among those we tested in the detection function model might have influenced porpoise behaviour or detectability during the surveys. Ongoing investigations aim to explore aspects of environmental variation that may be contributing to changes in detectability.

### 4.3 Relationships with environmental variables

Hotspots of primary productivity and areas characterised by prey aggregating processes are often associated with the predictable occurrence of marine predators (Cox et al., 2018; Davis et al., 2002; Forney et al., 2015), including harbour porpoises (Gilles et al., 2011, 2016; Johnston et al., 2005; Scott et al., 2010). Fluctuations in porpoise presence in relation to SST were suggested to reflect changes in prey abundance caused by regular upwelling events along the northwestern coast of Spain (Díaz López & Methion, 2018). Our study revealed a higher probability of sighting an animal at lower temperatures, in line with other studies, likely in association with the presence of preferred prey species (Rekdahl et al., 2023; Wingfield et al., 2017). Despite findings of harbour porpoises associated with cooler water temperatures, this species has also been reported to inhabit areas with warmer SSTs, such as the North Sea (Gilles et al., 2011). The relationship between SST and harbour porpoise presence seems to vary among populations.

CHLA, fronts, SSH, MLD, which are common oceanographic measures of mesoscale activities (Davis et al., 2002; Gilles et al., 2016; Virgili et al., 2019), are also often used as proxies for prey abundance resulting from primary production and nutrient‐rich waters (Goetsch et al., 2023; Mann & Lazier, 2006). Harbour porpoises in the German Bight aggregate along steep gradients of CHLA concentration (Gilles et al., 2011). In the northwestern Atlantic, peaks in porpoise occurrence at high CHLA concentrations of 4.5–7.4 mg m^−3^ were interpreted as indicative of greater numbers of forage fish (Wingfield et al., 2017). Mixed layer depth has also been reported to be an important predictor of the abundance and size of forage fish aggregations, thus reflecting prey availability to top predators (Goetsch et al., 2023). Equally, areas with high primary productivity are often found in association with fronts (Polovina et al., 2001), which can promote biological production and prey aggregation (Franks, 1992; Gilles et al., 2016; Johnston et al., 2005; Mann & Lazier, 2006). Overall, our study has demonstrated that these proxies for harbour porpoise prey aggregation are important covariates to include at suitable scales when modelling their distribution.

## 5 CONCLUSION

Our study presents a procedure to identify the most suitable resolution of modelled and remotely sensed oceanographic variables to model harbour porpoise distribution. The best spatial and temporal scales for each variable that were selected here were inconsistent with the resolutions used to model harbour porpoise habitat in other studies. Differences in spatial scale mostly emerged because previous studies have used predictor variables at a single resolution, typically fine‐scale (Gilles et al., 2011; Rekdahl et al., 2023; Wingfield et al., 2017, but see Gilles et al., 2016), but could also be attributed to different physical processes occurring in a particular study area (Johnston et al., 2005). The environmental variables commonly used in those studies were also averaged over longer time scales (e.g. a week), which is in line with our findings. Future studies modelling seasonal distribution of harbour porpoise using modelled and remotely sensed oceanographic variables should follow the procedure established here. This approach will allow for a better understanding of the broad‐ and fine‐scale processes driving seasonal habitat selection of harbour porpoises. In general, investigating the effects of scale in seasonal habitat models is critical when assessing predator distribution, particularly during a time of climate uncertainty and given the urgent need to inform conservation and management efforts.

## AUTHOR CONTRIBUTIONS

**Tiffany Goh:** Conceptualization (equal); data curation (lead); formal analysis (equal); investigation (lead); methodology (equal); project administration (equal); writing – original draft (lead). **Mark Jessopp:** Conceptualization (equal); data curation (supporting); formal analysis (supporting); funding acquisition (equal); investigation (supporting); methodology (supporting); project administration (equal); supervision (equal); writing – review and editing (equal). **Emer Rogan:** Conceptualization (equal); data curation (supporting); formal analysis (supporting); funding acquisition (equal); investigation (supporting); methodology (supporting); project administration (equal); supervision (equal); writing – review and editing (equal). **Enrico Pirotta:** Conceptualization (equal); data curation (supporting); formal analysis (equal); investigation (supporting); methodology (equal); project administration (equal); supervision (equal); writing – review and editing (equal).

## FUNDING INFORMATION

Department of Environment, Climate and Communications (DECC) in partnership with the Department of Housing, Local Government and Heritage (DHLGH), the Sustainable Energy Authority of Ireland (SEAI) and Geological Survey Ireland (GSI).

## CONFLICT OF INTEREST STATEMENT

The authors declare no conflict of interest.

## Supporting information


Data S1

## Data Availability

Harbour porpoise presence and absence data with associated values of each oceanographic covariate are available on Dryad (https://doi.org/10.5061/dryad.s7h44j1dv). Data from all modelled and remotely sensed variables were downloaded from https://data.marine.copernicus.eu/.
