# Supplementary material for: A matter of scale: Identifying the best spatial and temporal scale of environmental variables to model the distribution of a small cetacean
Source: Ecol Evol. 2024 Aug 6;14(8):e70102. doi: 10.1002/ece3.70102 (PMC11301658; doi:10.1002/ece3.70102)
Supplement: Supplementary file 1 — Data S1 [file ECE3-14-e70102-s001.docx]

**A matter of scale: Identifying the best spatial and temporal scale of environmental variables to model the distribution of a small cetacean**

**Supplementary Figures**

**S1.** Plots for raw and log-transformed chlorophyll-a data. chla: Chlorophyll-a, spatial scale includes 7, 21 and 42 km, d: daily, m: monthly, p: survey period.

**S2.** Plots for raw and log-transformed mixed layer depth data. mld: mixed layer depth, spatial scale includes 7, 21 and 42 km, d: daily, m: monthly, p: survey period.

**S3.** Hazard rate detection function plot for 2016.

**S4.** Hazard rate detection function plot for 2021.

**S5.** Half-normal detection function plot for 2022.

**S6.** 5 km spatial resolution of sea surface temperature plotted across the survey period of 2016. This resolution was used as the finest scale of this study.

**
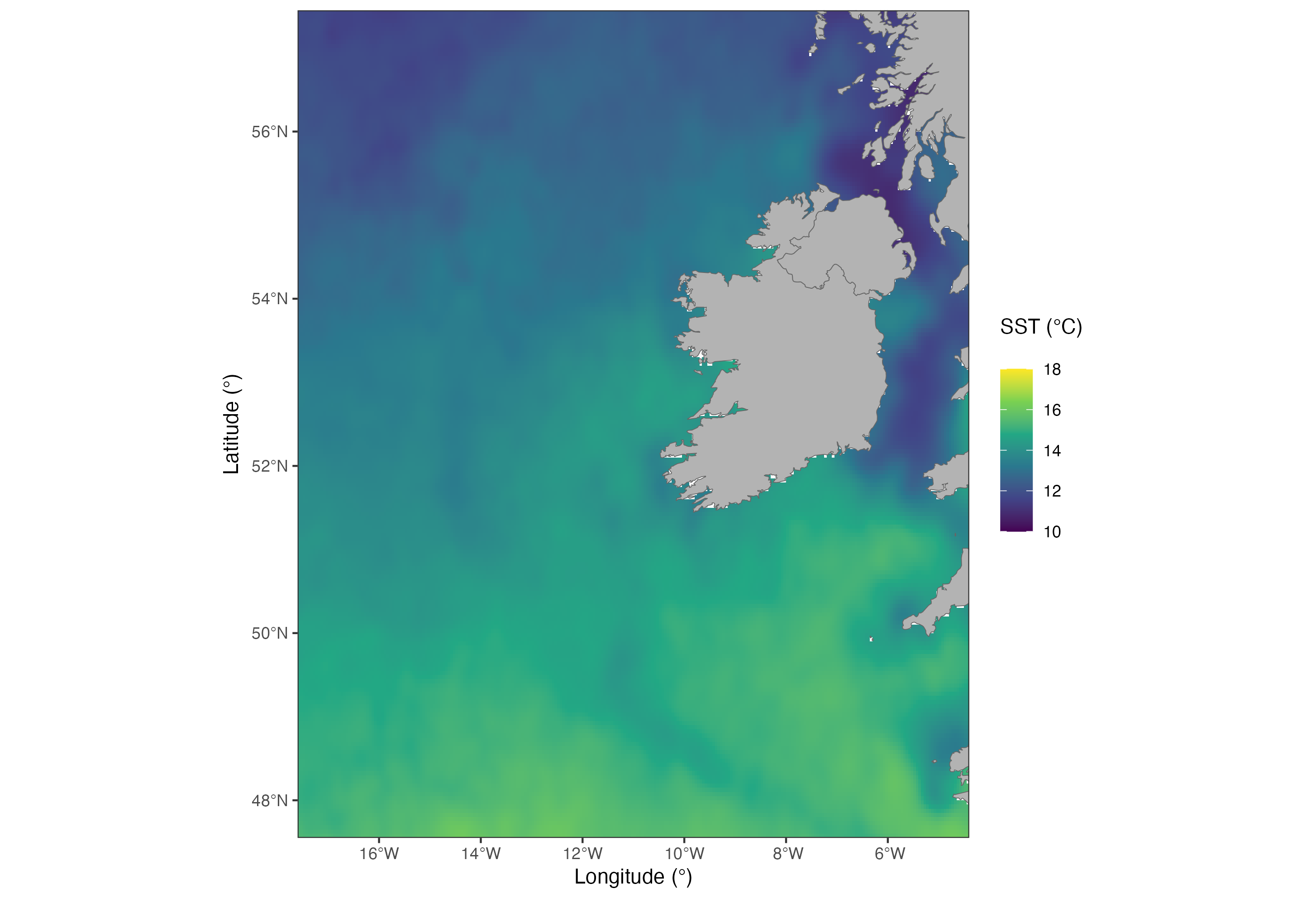
**

**S7.** 5 km spatial resolution of thermal fronts plotted across the survey period of 2016. This resolution was used as the finest scale of this study.

**
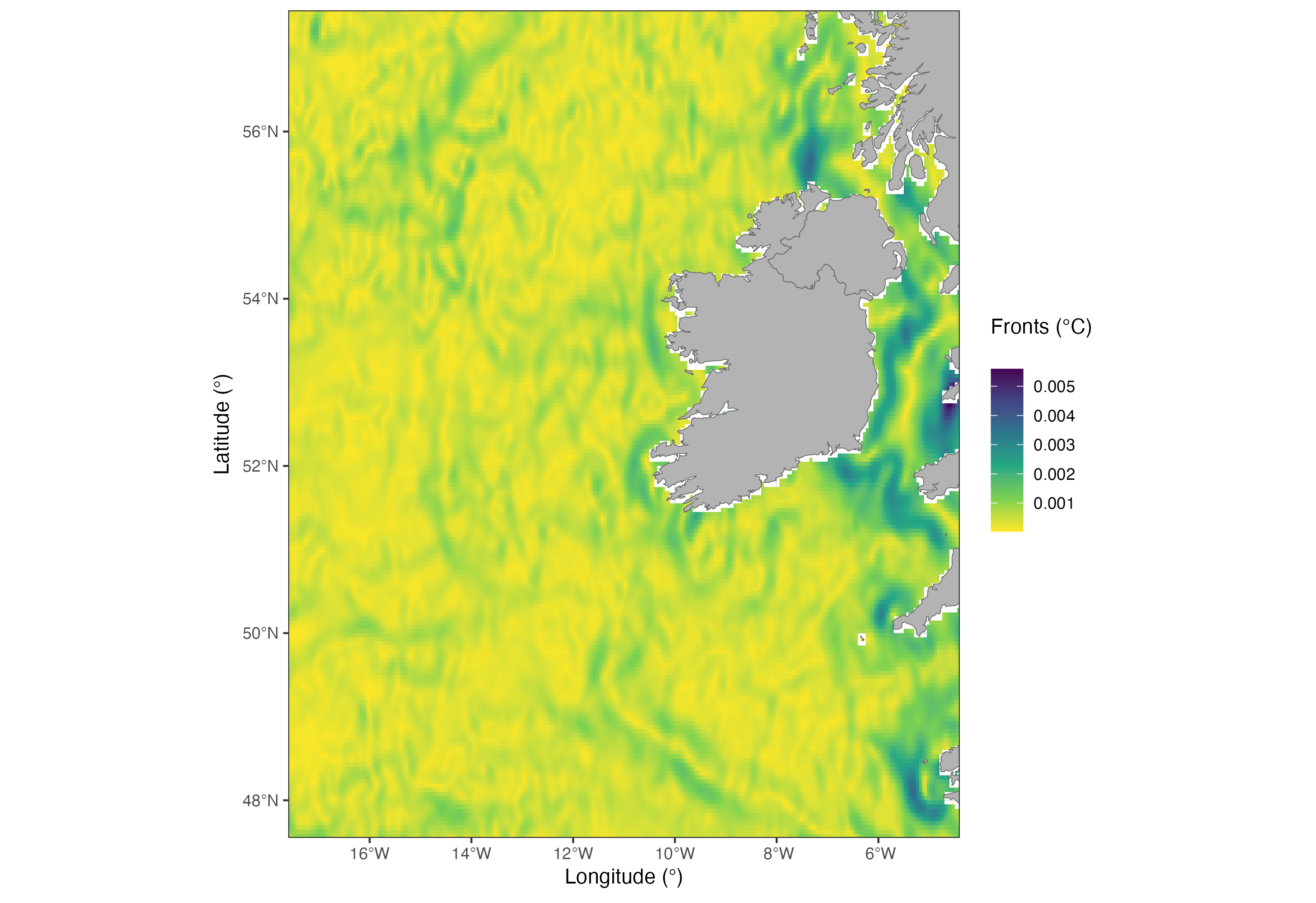
**

**S8.** 7 km spatial resolution of chlorophyll-a plotted across the survey period of 2016. This resolution was defined as the finest scale of this study (5 km).

**
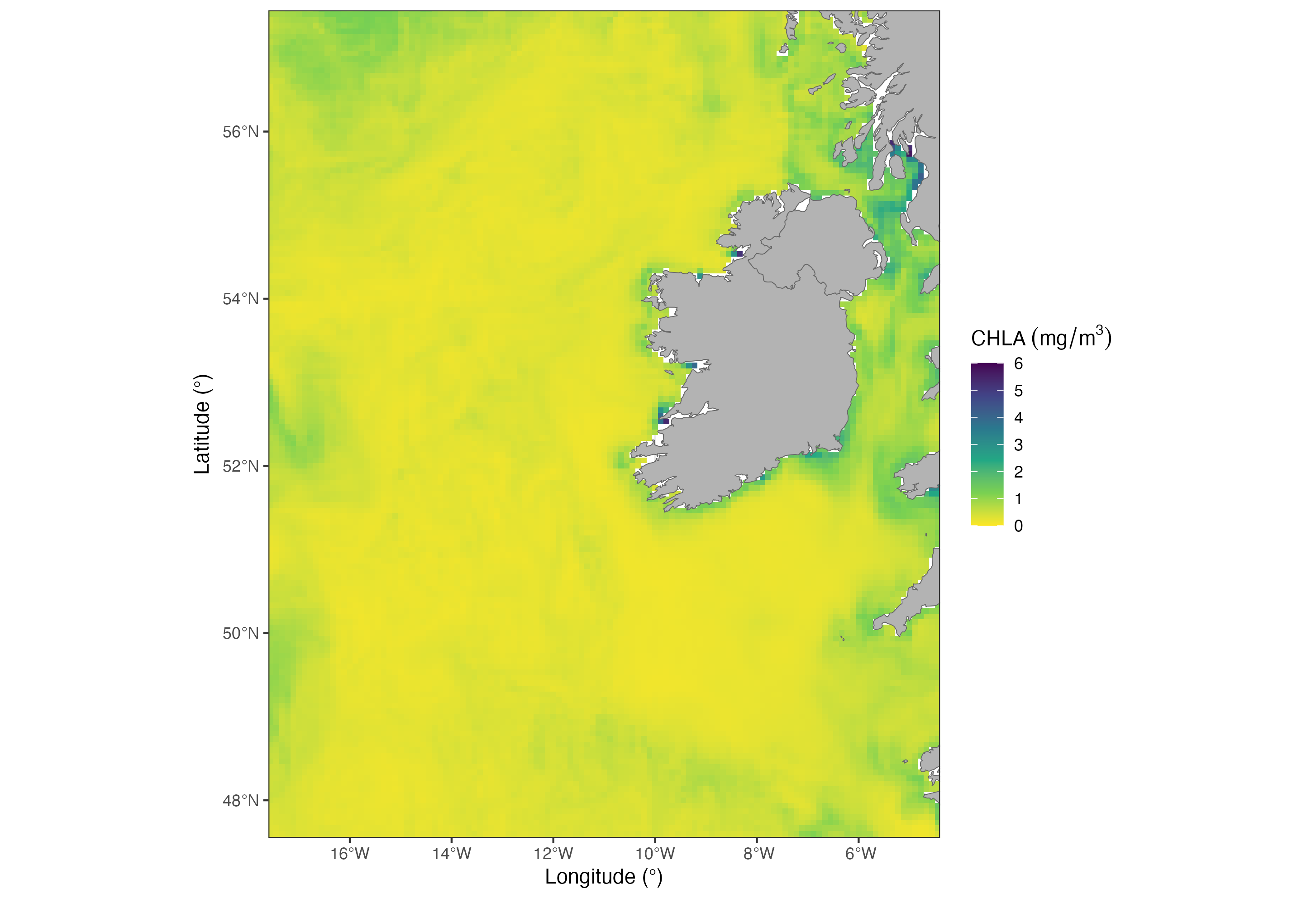
**

**S9.** 7 km spatial resolution of sea surface height plotted across the survey period of 2016. This resolution was defined as the finest scale of this study (5 km).

**
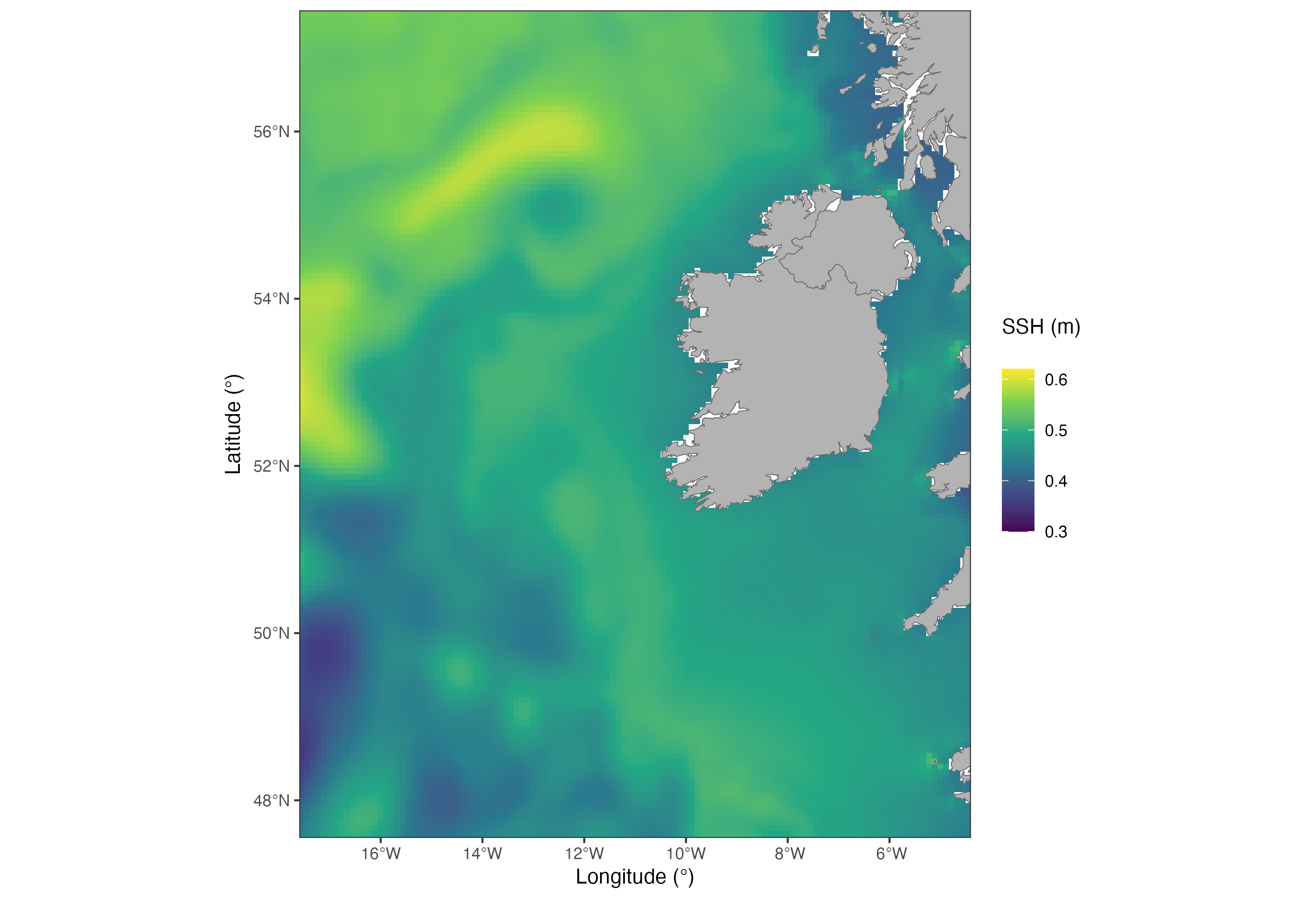
**

**S10.** 7 km spatial resolution of mixed layer depth plotted across the survey period of 2016. This resolution was defined as the finest scale of this study (5 km).

**
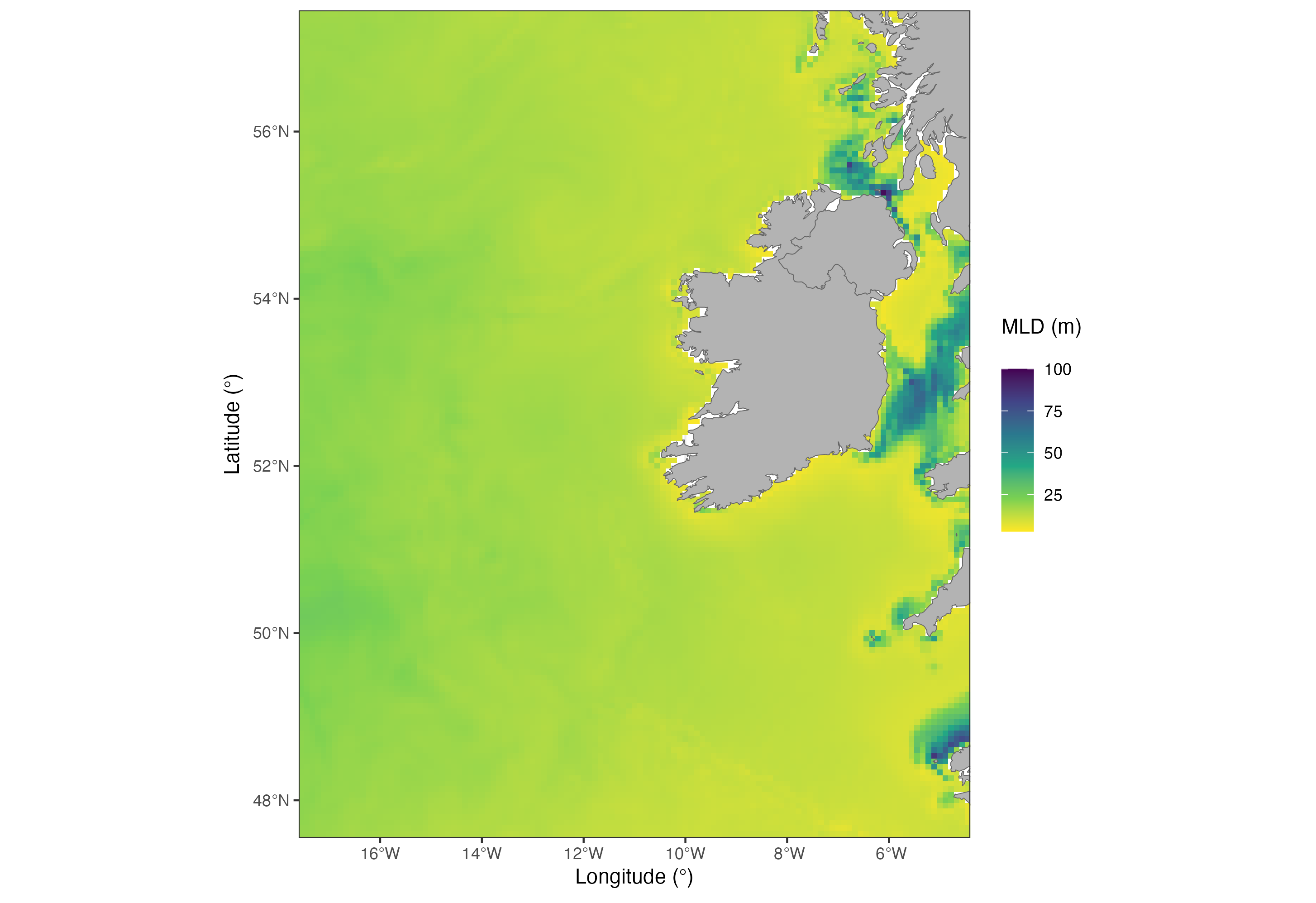
**

**S11.** 7 km spatial resolution of salinity plotted across the survey period of 2016. This resolution was defined as the finest scale of this study (5 km).

**
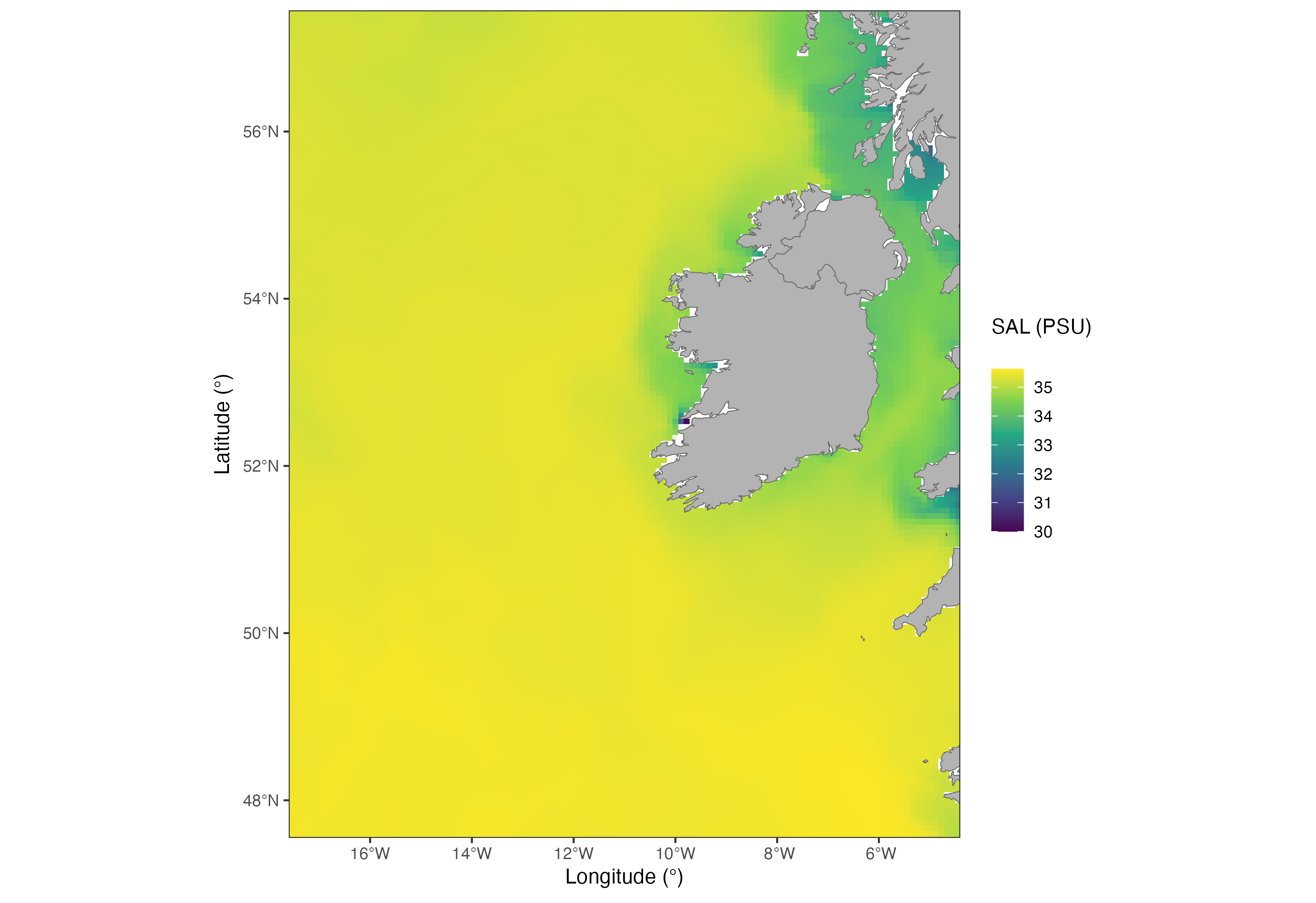
**

**S12.** Map of 2016 transect lines overlaid with a 4 x 4 km grid.

**
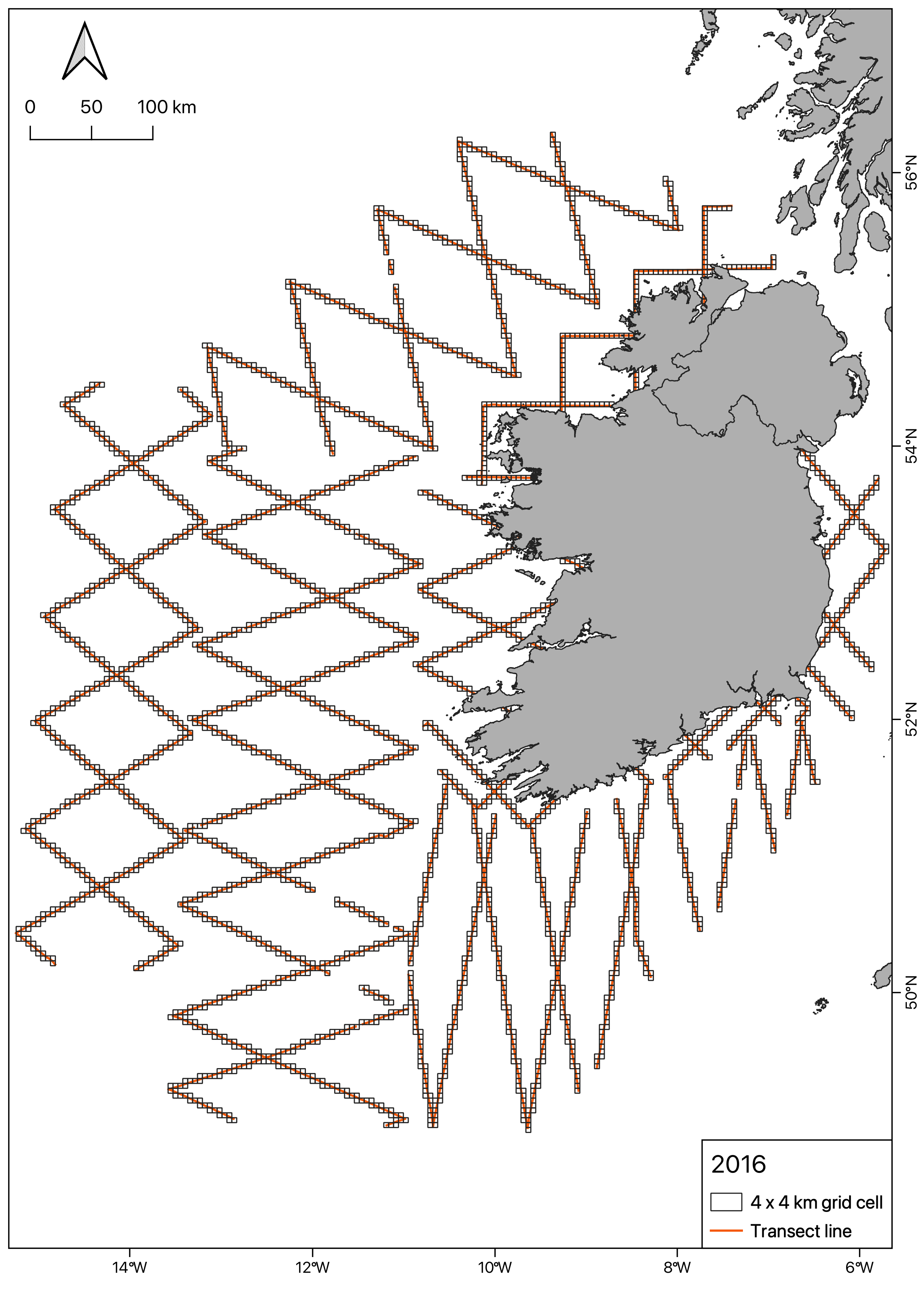
**

**S13.** Map of 2021 transect lines overlaid with a 4 x 4 km grid.

**
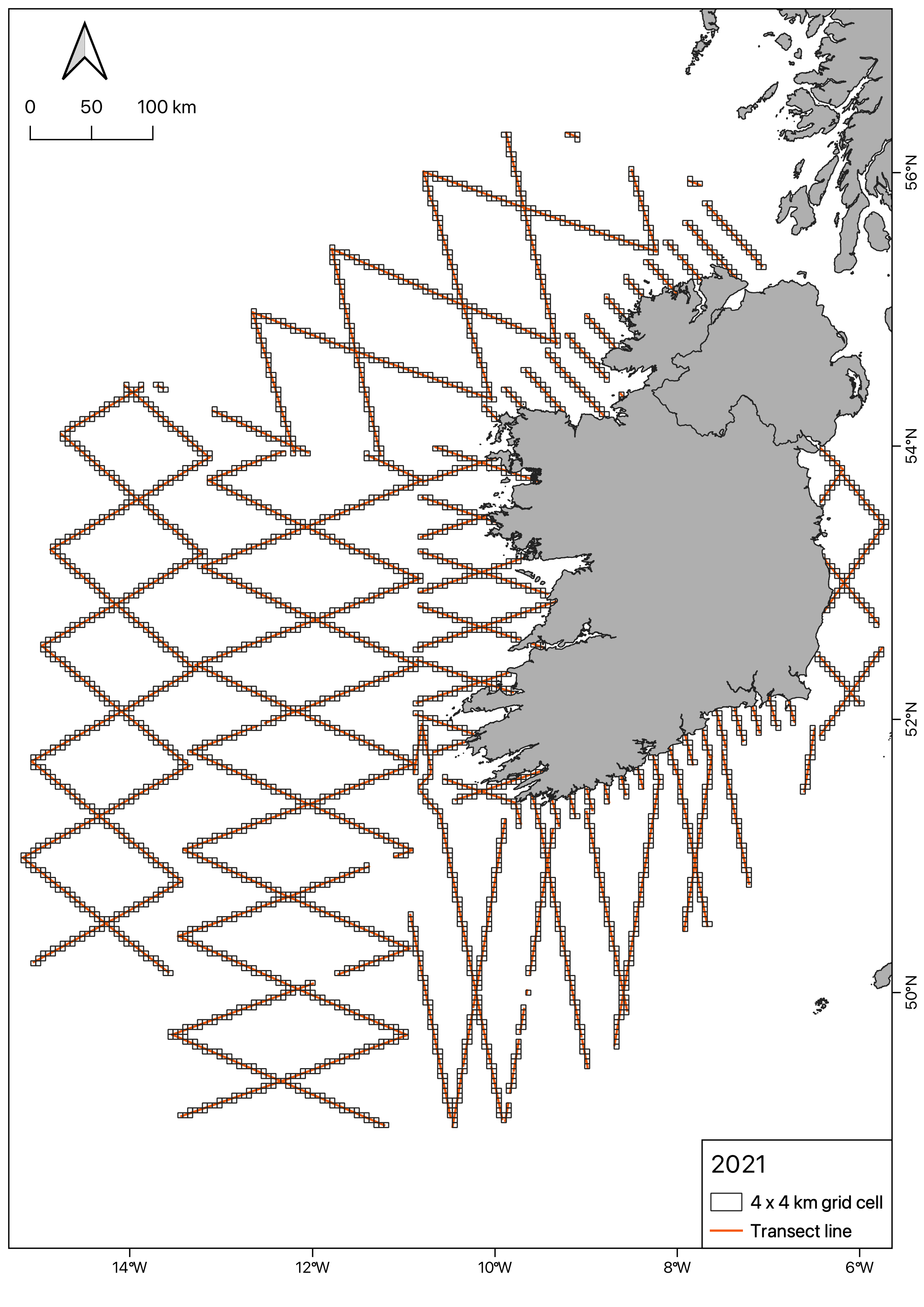
**

**S14.** Map of 2022 transect lines overlaid with a 4 x 4 km grid.

**
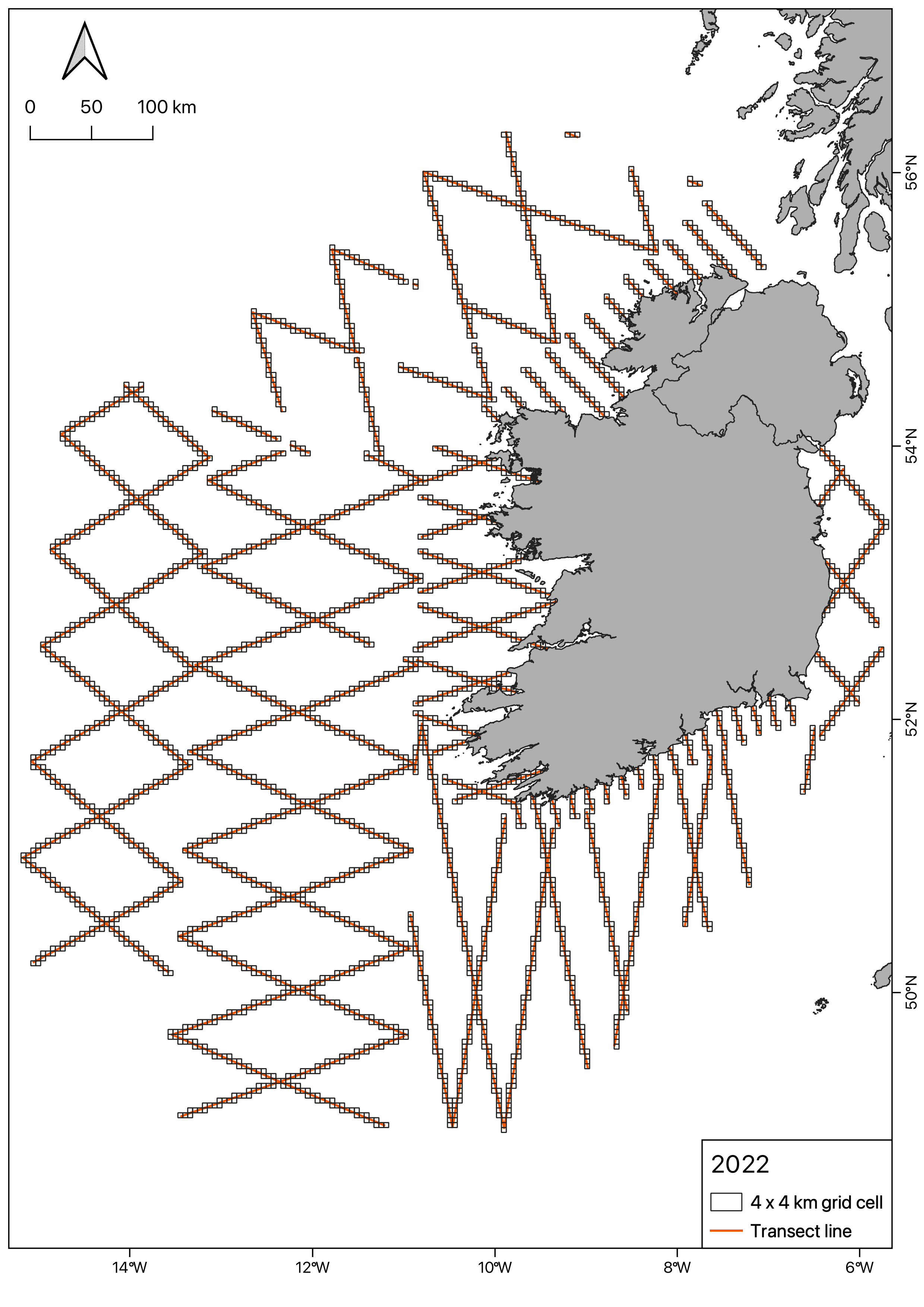
**

**Supplementary Table**

**S1.** Table of best-fitting models for each oceanographic variable after model selection and validation. 40d = 40 km spatial and daily temporal resolution, 40m = 40 km spatial and monthly temporal resolution, 40p = 40 km spatial and survey period temporal resolution, 5p = 5km spatial and survey period temporal resolution.

| Oceanographic variables | Best-fitting model |
| --- | --- |
| Sea surface temperature (SST) | year + depth + effort + SST40d |
| Chlorophyll-a (CHLA) | year + depth + effort + CHLA40d |
| Sea surface height (SSH) | year + depth + effort + SSH40m |
| Thermal fronts (Fronts) | year + depth + effort + Fronts40m |
| Salinity (SAL) | year + depth + effort + SAL40p |
| Mixed layer depth (MLD) | year + depth + effort + MLD5p |

**S2.** Comparison between best (top) and worst (bottom) fitting models for each oceanographic variable, and associated goodness-of-fit measures. Models include depth, effort, year, and each oceanographic variable at the specified spatio-temporal resolution. VIF = Variance Inflation Factor, ROC = Receiving Operating Characteristic, 40d = 40 km spatial and daily temporal resolution, 40m = 40 km spatial and monthly temporal resolution, 40p = 40 km spatial and survey period temporal resolution, 5p = 5km spatial and survey period temporal resolution.

|  | VIF | Concurvity | *p*-value of smooth terms | Deviance explained (%) | Area under ROC curve | Confusion matrix statistics | | |
| --- | --- | --- | --- | --- | --- | --- | --- | --- |
|  |  |  |  |  |  | **Accuracy (%)** | **Sensitivity (%)** | **Specificity (%)** |
| SST40d | 1.0 | 0.66 | <0.0001 | 25.8 | 0.9 | 76 | 75 | 93 |
| Depth | 1.0 | 0.20 | <0.0001 |  |  |  |  |  |
| Effort | 1.0 | 0.33 | <0.0001 |  |  |  |  |  |
|  |  |  |  |  |  |  |  |  |
| SST5p | 1.0 | 0.67 | <0.0001 | 23.2 | 0.89 | 70 | 70 | 95 |
| Depth | 1.0 | 0.15 | <0.0001 |  |  |  |  |  |
| Effort | 1.0 | 0.33 | <0.0001 |  |  |  |  |  |
|  |  |  |  |  |  |  |  |  |
| CHLA40d | 1.0 | 0.52 | <0.0001 | 24.7 | 0.9 | 77 | 77 | 90 |
| Depth | 1.0 | 0.13 | <0.0001 |  |  |  |  |  |
| Effort | 1.0 | 0.33 | <0.0001 |  |  |  |  |  |
|  |  |  |  |  |  |  |  |  |
| CHLA5p | 1.0 | 0.69 | < 0.05 | 23.3 | 0.89 | 79 | 79 | 87 |
| Depth | 1.0 | 0.21 | <0.0001 |  |  |  |  |  |
| Effort | 1.0 | 0.33 | <0.0001 |  |  |  |  |  |
|  |  |  |  |  |  |  |  |  |
| SSH40m | 1.1 | 0.39 | <0.0001 | 25.4 | 0.9 | 77 | 77 | 92 |
| Depth | 1.1 | 0.24 | <0.0001 |  |  |  |  |  |
| Effort | 1.0 | 0.33 | <0.0001 |  |  |  |  |  |
|  |  |  |  |  |  |  |  |  |
| SSH5d | 1.0 | 0.26 | 0.905 | 23.1 | 0.89 | 76 | 76 | 90 |
| Depth | 1.0 | 0.14 | <0.0001 |  |  |  |  |  |
| Effort | 1.0 | 0.33 | <0.0001 |  |  |  |  |  |
|  |  |  |  |  |  |  |  |  |
| Fronts40m | 1.1 | 0.68 | <0.0001 | 25.1 | 0.9 | 78 | 77 | 92 |
| Depth | 1.1 | 0.37 | <0.0001 |  |  |  |  |  |
| Effort | 1.0 | 0.34 | <0.0001 |  |  |  |  |  |
|  |  |  |  |  |  |  |  |  |
| Fronts5d | 1.0 | 0.31 | <0.01 | 24.6 | 0.9 | 74 | 74 | 94 |
| Depth | 1.0 | 0.16 | <0.0001 |  |  |  |  |  |
| Effort | 1.0 | 0.34 | <0.0001 |  |  |  |  |  |
|  |  |  |  |  |  |  |  |  |
| SAL40p | 1.2 | 0.68 | <0.0001 | 26.5 | 0.9 | 78 | 78 | 90 |
| Depth | 1.2 | 0.67 | <0.0001 |  |  |  |  |  |
| Effort | 1.0 | 0.33 | <0.0001 |  |  |  |  |  |
|  |  |  |  |  |  |  |  |  |
| SAL20m | 1.2 | 0.68 | <0.0001 |  |  |  |  |  |
| Depth | 1.2 | 0.66 | <0.0001 | 25.5 | 0.9 | 79 | 79 | 90 |
| Effort | 1.0 | 0.33 | <0.0001 |  |  |  |  |  |
|  |  |  |  |  |  |  |  |  |
| MLD5p | 1.2 | 0.54 | <0.0001 | 24.2 | 0.9 | 75 | 74 | 92 |
| Depth | 1.2 | 0.48 | <0.0001 |  |  |  |  |  |
| Effort | 1.0 | 0.33 | <0.0001 |  |  |  |  |  |
|  |  |  |  |  |  |  |  |  |
| MLD20p | 1.2 | 0.51 | 0.219 |  |  |  |  |  |
| Depth | 1.2 | 0.45 | <0.0001 | 23.1 | 0.89 | 76 | 76 | 90 |
